# Hyperbaric Oxygen Improves Cognitive Impairment Induced by Hypoxia via Upregulating the Expression of Oleic Acid and MBOAT2 of Mice

**DOI:** 10.3390/antiox13111320

**Published:** 2024-10-29

**Authors:** Zhen Li, Jun Fu, Kaiyuan Jiang, Jie Gao, Yuejun Guo, Chen Li, Liangcai Zhao, Jutaek Nam, Hongchang Gao

**Affiliations:** 1 School of Pharmaceutical Sciences, Oujiang Laboratory (Zhejiang Lab for Regenerative Medicine, Vision and Brain Health), Institute of Metabonomics & Medical NMR, Wenzhou Medical University, Wenzhou 325035, Chinafujun6866@wmu.edu.cn (J.F.); jiangkyxf@wmu.edu.cn (K.J.); lichen2zh@wmu.edu.cn (C.L.); zhaoliangcai@wmu.edu.cn (L.Z.); 2 College of Pharmacy, Chonnam National University, Gwangju 61186, Republic of Korea; 3 Innocation Academy of Testing Technology, Research and Experiment Center, Wenzhou Medical University, Wenzhou 325035, China

**Keywords:** hyperbaric oxygen, cognitive impairment, ferroptosis, membrane phospholipid remodeling, MBOAT2, oleic acid

## Abstract

Cognitive impairment (CI) causes severe impairment of brain function and quality of life of patients, which brings a great burden to society. Cerebral hypoxia is an important factor in the pathogenesis of CI. Hyperbaric oxygen (HBO) therapy may mitigate hypoxia-induced CI, but its efficacy and mechanisms are not fully understood. In this study, a mice model of CI induced by hypoxia environment was established, then behavioral tests, pathological examination, metabolomic and lipidomic analyses, and molecular biology were used to assess the impact of HBO on hypoxia-induced CI. HBO was found to alleviate CI and pathological damage of hypoxia mice. Metabolomic, lipidomic, and molecular biology analyses showed that HBO increased the levels of oleic acid (OA) and membrane-bound O-acyltransferase 2 (MBOAT2), thereby altering the composition of membrane phospholipids (PLs) and reducing hypoxia-induced neuronal ferroptosis (FPT) to interfere with cognitive function in mice. In vitro experiments confirmed that OA and MBOAT2 led to membrane PL remodeling in a mutually dependent manner, affecting cell resistance to hypoxia-FPT. The results emphasized the combined effect value of OA and MBOAT2 in HBO for hypoxia-induced CI, and provided a novel perspective for the treatment of CI by HBO.

## 1. Introduction

Cognitive impairment (CI) is characterized by poor attention, causal reasoning, learning, and memory, impacting 3–19% of those over 65 worldwide [1]. In addition to being prevalent in aging and aging-related neurodegenerative diseases, CI is also associated with multiple diseases, making CI an urgent global health issue. Actually, high altitude, respiratory diseases leading to hypoxemia affect cognitive function. Data indicated a 22% CI prevalence and 11% dementia prevalence in seniors at high altitudes, with significant effects observed above 4000 m [2,3]. Currently, there is substantial evidence indicating that high altitude is a contributing factor to the high prevalence of CI [4,5,6,7,8,9,10,11], and research has been conducted on the mechanisms and treatment options related to this issue. Post-Acute Respiratory Distress Syndrome (ARDS) is a devastating condition that affects individuals, with a 70–100% experiencing CI at discharge, decreasing to 20% after five years, heavily impacting quality of life [12,13]. Heart disease, carbon monoxide, and traumatic brain injury can also contribute to CI by reducing cerebral oxygen [14,15,16,17]. These diseases and environmental and behavioral factors all lead to CI, and a common feature is physiological stressors related to hypoxia, which are particularly toxic to brain regions that require high oxygen consumption [18,19]. Hypoxia, from any source, posed significant risks to oxygen-dependent brain areas. Therefore, understanding the impact of hypoxia on the central nervous system (CNS) and its molecular mechanisms is vital for patient care and life quality.

Numerous studies have provided substantial evidence supporting the concept that hypoxia induces oxidative stress. Exposure to hypoxia has been shown to elevate reactive oxygen species (ROS) levels and oxidized glutathione (GSH), indicating that hypoxia can induce oxidative stress [20]. Both in vivo and in vitro experiments have demonstrated that hypoxic environments promote an elevation in ROS and malondialdehyde (MDA) levels [21,22]. Ferroptosis (FPT) is a distinctive cell death pathway triggered by oxidative stress, characterized by lipid peroxidation and depletion of GSH [23,24]. Oxidative stress and FPT play crucial roles in the development and progression of pathology related to hypoxia-related diseases [25,26,27,28], emphasizing the importance of gaining deeper insights into molecular changes associated with brain hypoxia-induced FPT.

Hyperbaric oxygen (HBO) therapy involves administering oxygen at pressures higher than atmospheric levels for medical treatment. It is effective for approved clinical conditions like carbon monoxide poisoning, wounds, air embolism, and decompression sickness [29,30]. Currently, there are 13 FDA-approved indications for this therapy [31], but off-label applications, particularly for cognitive and neurological health improvements post-brain injury or stroke, are increasing [14,32]. Although not yet established by the FDA as a treatment for ischemic hypoxic encephalopathy, numerous studies have demonstrated a protective effect of HBO therapy [33,34,35,36,37,38,39,40,41,42], including some human trials [34,35,40,41]. A systematic review of 20 trials investigated the clinical effectiveness of HBO treatment for neonatal hypoxic-ischaemic encephalopathy and found that it possibly reduced mortality and neurological sequelae in term neonates with this condition [43]. This opens up new avenues in the treatment of neurological disorders and improving cognition and brain metabolism in CI patients. One of the main mechanisms of HBO is that it can increase the partial pressure of oxygen in the blood and tissues [44]. This increases the amount of oxygen entering the plasma by five to ten times and reaching the oxygen-deprived tissues. Despite its use, the exact mechanisms of HBO remain unclear.

In the current study, a hypoxia environment was employed as the modeling condition for CI of mice. Subsequently, the impact of HBO on CI was investigated using this model. Various assessments were conducted, including behavioral tests, pathological examination, metabolomics and lipidomics analyses and molecular biology techniques, to evaluate the effects of HBO on hypoxia-induced CI and its potential regulatory pathways. After the key metabolites and genes were identified, lipidomics analyses and molecular biology methods were utilized to further clarify the underlying mechanisms of their intervention.

## 2. Materials and Methods

### 2.1. Animals and Treatment

Seven-week-old male C57BL/6 mice (*n* = 30) were divided into three groups: control (CON, atmospheric oxygen), hypoxia (10% O_2_ for 7 days), and HBO (following 7 days of hypoxia, treated with 100% O_2_ at 2 ATA for 1 h daily for 5 days). All procedures were ethically approved by Wenzhou Medical University’s Ethics Committee (No. wydw2024-0097) and utilized a ProOx-850 high and low pressure oxygen chamber (Tarzan tech, Shanghai, China). The treatment regimen aligned with previous studies [14,45,46,47].

### 2.2. Behavioral Tests

For the Y-maze test, mice were placed in the center of the maze (L = 30 cm, W = 8 cm, H = 15 cm) and allowed to explore for 6 min. The total number of runs and alternative behavior were recorded and analyzed. The cognitive and memory function of mice was evaluated by water maze test (MWT) as previously described [48]. The mice were subjected to training tests for four consecutive days. On day 5, the platform was removed and the mice were placed in the water for a 65 s probe test at the farthest quadrant from the hidden platform. The data and video were monitored by the overhead camera and measured by MWT-100 Morris software (Taimeng Software Co., Ltd., Chengdu, China).

### 2.3. Nissl Staining and Immunofluorescence (IF)

The brain sections were soaked in Nissl staining solution (Beyotime Biotechnology, Nanjing, China) and dehydrated in 95% ethanol, dimethylbenzene. As for IF, the brain sections were exposed to the primary antibodies against NeuN (Proteintech, Wuhan, China, 66836-1-Ig, 1:200) at 4 °C overnight, then incubated with goat anti-IgG (Bioworld, Nanjing, China, CI36131, 500:1) for 1 h at 37 °C. The staining was obtained using an Olympus BX53 microscope (Olympus, Shinjuku, Tokyo, Japan).

### 2.4. Untargeted and Targeted Ultra-Performance Liquid Chromatography-Tandem Mass Spectrometry (UHPLC-MS) Analysis

MeOH/water and ACN/water were used to obtain polar compounds from the sample. IPA was added to get lipid compounds. Sample analysis was performed on a SHIMADZU CBM-30A Lite LC system (Shimadzu Corpoion, Kyoto, Japan) coupled with API 6600 Triple TOF (AB SCIEX, Foster City, CA, USA) detector. Principal component analysis (PCA) and supervised orthogonal partial least squares discriminant analysis (OPLS-DA) were conducted to evaluate model performance, with R2Y and Q2 indicating model fit and predictability, respectively. Univariate analysis, including a Student’s *t*-test, was performed through omicshare.com to identify significant metabolites based on *p* < 0.05, VIP > 1.0, and fold change (FC) < 0.5 or >2. The metabolites were further identified through MS-DIAL based on precise molecular weight and signature fragments and validated through HMDB 4.0. The fatty acids (FAs) were quantitatively analyzed by API 6500 Q-TRAP mass spectrometer (AB SCIEX, Foster City, CA, USA). The levels of phospholipids (PLs) were detected by semi-targeted lipidomics multi-reaction monitoring (MRM). Heat maps and volcano plot were drawn using the cluster Profiler package (version 4.8.3) in R software. Enrichment analysis of KEGG pathways were performed on Metaboanalyst 5.0. Venn diagrams were created on omicshare.com (accessed on 20 June 2024).

### 2.5. Quantitative Real-Time Polymerase Chain Reaction (qRT-PCR) Analysis

The total RNA from prefrontal cortex tissues was extracted using TRIzol (Takara, Kusatsu, Japan) and converted to cDNA with HiScript III RT SuperMix (Vazyme, Nanjing, China). qRT-PCR analysis was performed using an SYBR Green Master mix (Vazyme, China) with primers for MBOAT2, ACSL3, AGPAT3, LPCAT3, MBOAT7, and ACSL4 listed in Table S1.

### 2.6. Detection of Ferrous Ion Content (Fe^2+^), MDA and GSH Levels

The levels of Fe^2+^, MDA and GSH were detected in sample as per the manufacturer’s instructions (Fe^2+^, MDA, Solarbio, Beijing, China; GSH, Njjcbio, Nanjing, China), then measured the absorbance value using a microplate reader (Labserv K3 enzyme marker, Thermo Fisher Scientific, Cleveland, OH, USA).

### 2.7. Cell Culture and Cell Viability Assay

The SH-SYSY cells selected in this study were human neuroblastoma cells purchased from the American Culture Preservation Center (ATCC). SH-SY5Y cells were cultured in DMEM/F-12 (Biosharp, Heifei, China) with 10% fetal bovine serum (Gimini, Saltillo, Mexico) and 1% Penicillin-Streptomycin (Solarbio, China) at 5% CO_2_, 37 °C. Cells were seeded in 96-well plates (2 × 10^3^ cells/well) and incubated overnight. After attachment, drugs were added before exposing the cells to hypoxia (1% O_2_, 5% CO_2_). Cell viability was measured using the CCK-8 assay (GLPBIO Biotechnology, Montclair, CA, USA), and absorbance at 450 nm was recorded.

### 2.8. Western Blot Analysis

Samples were dissolved in a lysis buffer (RIPA: phosphatase protease inhibitor: protease inhibitor = 100:1:1). The protein was separated with SDS-PAGE gel and transferred to PVDF membranes, and incubated overnight at 4 °C with the primary antibodies against: Glutathione Peroxidase 4 (GPX4) (proteintech), Solute carrier family 7 member 11 (SLC7A11) (proteintech), Postsynaptic Density 93 (PSD93) (HUABIO, Woburn, MA, USA). Afterward, membranes were incubated with HRP-conjugated secondary antibodies for 1 h at room temperature. The proteins were visualized using the ChemiDoc™MP imaging system (BIO-RAD, Hercules, CA, USA) with a chemiluminescent substrate kit (New Cell & Molecular Biotech, Shanghai, China).

### 2.9. The Detection of Lipid Peroxidation in Cells

Harvested at the density of 10 × 10^6^, cells were stained with Bodipy (BODIPY™ 581/591 C11, Invitrogen, St. Bend, OR, USA) to detect lipid peroxidation level. The signals were collected by flow cytometry (CytoFLEX, Beckman Coulter, Chaska, MN, USA).

### 2.10. Statistical Analysis

Statistical analysis was performed using GraphPad Prism version 8.2.1 and SPSS software version 22.0. For each figure, *n* = the number of independent biological replicates. The sample sizes used in the research were sufficient to yield statistical significance [49,50], and this was also reflected in other studies [27,51,52]. Differences between two treatment groups were assessed using two-tailed, unpaired Student’s *t* test. Differences among >2 groups with only one variable were assessed using one-way ANOVA, followed by Tukey’s post-hoc test for multiple comparisons. Significant differences emerging from the above tests are indicated in the figures by * *p* < 0.05, ** *p* < 0.01, *** *p* < 0.001. Notable non-significant differences are indicated in the figures by “ns”.

## 3. Results

### 3.1. HBO Improved CI and Pathological Changes Induced by Hypoxia in Mice

**Figure 1:**
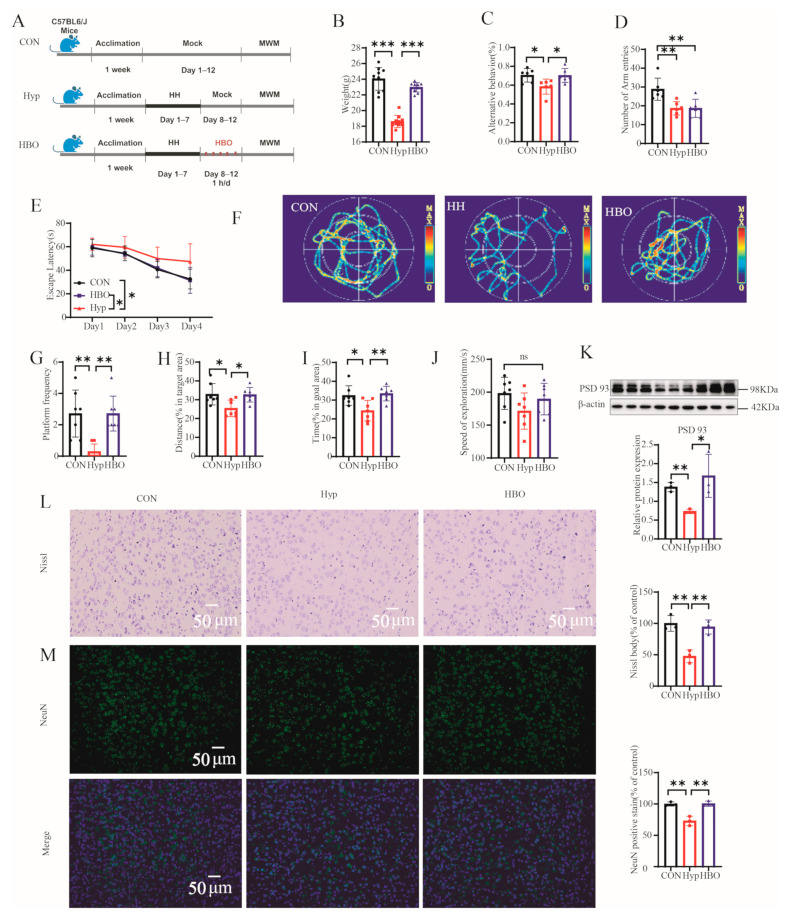
(**A**) Experimental timeline describing the time of exposing to CON, Hypoxia or HBO in all three groups (n = 10). (**B**) The weight of mice (n = 10). (**C**) Y maze spontaneous alternation. (**D**) The total number of arm entries of the Y maze (n = 6). (**E**) The escape latency. (**F**) The swimming trajectories. (**G**) The number of crossings over the original platform location of CON, Hypoxia, and HBO mice during the test period. The percentages of swimming (**H**) distance and (**I**) time in goal area. (**J**) The speed of exploration of CON, Hypoxia, and HBO mice during the test period (n = 7). (**K**) Western blots of PSD93 protein and corresponding quantitative results in the prefrontal cortex (n = 3). (**L**) Representative images of Nissl body staining (blue spots, 200×) and corresponding quantitative results in the prefrontal cortex (n = 3). Scale bar, 50 μm. (**M**) Representative images of NeuN staining (vivid green dots, 200×) and corresponding quantitative results in the prefrontal cortex (n = 3). The nucleus was stained with DAPI (blue color). Scale bar, 50 μm. Data are presented as mean ± standard error of the mean (SEM). The behavioral changes over time during the training period were analyzed by repeated measure ANOVA. Significant level: ns: not statistically significant, * *p* < 0.05; ** *p* < 0.01; *** *p* < 0.001.

To determine the impact of hypoxia and HBO on cognitive behavior, mice were given the Y-maze and MWT test to see the behavioral responses (Figure 1A). Hypoxia led to decreasing mice weight, which was restored by HBO (Figure 1B). In the Y-maze, hypoxia impaired working memory, as indicated by fewer correct alternations, but this was improved by HBO (Figure 1C). The total number of arm entries for hypoxia mice was the same as HBO mice, they all decreased compared to CON mice (Figure 1D). This suggested that the spatial working memory was disrupted in hypoxia group and improved in HBO group. However, the motivation for exploration decreased in both the hypoxia and HBO groups. In the MWT, hypoxia led to an increase in escape latency during the training period, but this was reversed by HBO treatment (Figure 1E). On the fifth day, HBO increased the number of crossings over the original platform location, reflecting an increase in the spatial memory of hypoxia mice (Figure 1F,G). Hypoxia resulted in a reduction of time and distance spent in the target quadrants, while HBO treatment increased these parameters (Figure 1H,I). Athletic ability was unaffected by varying oxygen levels, as swimming speeds remained similar across all groups (Figure 1J). Synapses are the fundamental structure for the transmission, processing and storage of information between neurons. The expression of PSD93 (Figure 1K), synapse-related marker, was significantly decreased in response to hypoxia exposure, then increased under HBO. HBO intervention also demonstrated improved neuronal health, as indicated by an increase in Nissl bodies and a higher intensity of NeuN positive staining in the prefrontal cortex of mice exposed to hypoxia (Figure 1L,M). These findings suggested that hypoxia impaired cognitive functions and neuronal health of mice, which can be mitigated by HBO treatment.

### 3.2. HBO Reversed Metabolic Disorders Induced by Hypoxia in Mice

**Figure 2:**
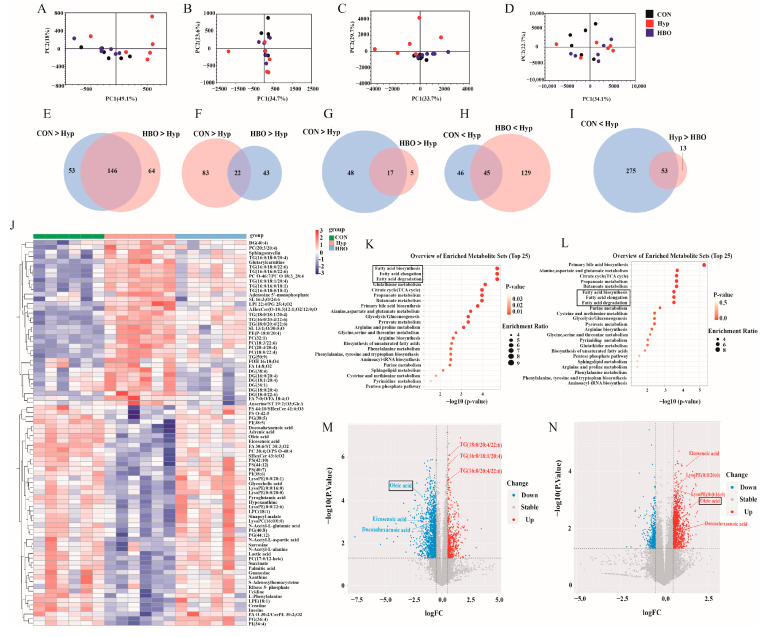
Hypoxia and HBO reprogrammed the cortical metabolome in mice. (**A**) Negative and (**B**) positive pattern of metabolic, (**C**) negative and (**D**) positive pattern of lipidic pattern changes in all three groups were analyzed by PCA (n = 6). Venn Diagram summarized the differential metabolites and lipids (**E**) negative and (**F**) positive pattern of metabolic (**G**,**H**) negative and (**I**) positive pattern of lipidic pattern changes in all three groups (n = 6). (**J**) The heat map showed relative levels of differential metabolites in the prefrontal cortex, with red indicating higher relative levels and blue indicating lower relative levels (n = 6). KEGG pathway enrichment analysis of the dysregulated pathways between (**K**) CON and Hypoxia group, (**L**) Hypoxia vs. HBO group. Volcano plot of all differentially expressed genes between (**M**) CON and Hypoxia group, (**N**) Hypoxia vs. HBO group. Pathways and metabolites with significant changes were annotated with black boxes.

To obtain a comprehensive understanding of metabolites that may contribute to host cognition regulation, untargeted metabolomic and lipidomic analyses of the frontal prefrontal cortex were performed in all groups. These obtained features were next analyzed using PCA and OPLS-DA. The quality control (QC) was compactly center-clustered in the PCA plot, indicating stable instrument reproducibility across different analytical runs (Figure S1A,D,G,J). PCA analysis revealed significant differences between samples, suggesting that cerebral metabolites and lipids were significantly altered, particularly between hypoxia and CON/HBO groups, which were more obvious in ESI-modes (Figure 2A–D). The OPLS-DA model demonstrated reliability with good fitting and predictive performance, ESI-modes were superior to ESI+ (Figure S1B,C,E,F,H,I,K,L). Subsequently, the important differentiated metabolites and lipids were identified using a Venn diagram which showing consistent trends in the hypoxia group compared to the CON and HBO groups (VIP > 1.0, *p* < 0.05) (Figure 2E–I). The multigroup heat map showed a total of 82 metabolites which identified as listed in Tables S2 and S3 (Figure 2J). KEGG pathway enrichment analysis highlighted significant changes in FA biosynthesis, elongation, and degradation pathways (Figure 2K,L). A volcano plot further illustrated these changes, with oleic acid (OA) notably decreasing in the hypoxia group and increasing after HBO exposure. OA was identified as a critical metabolite potentially influencing cognition.

### 3.3. HBO Reversed the Changes of OA and Membrane PLs Caused by Hypoxia in Mice

**Figure 3:**
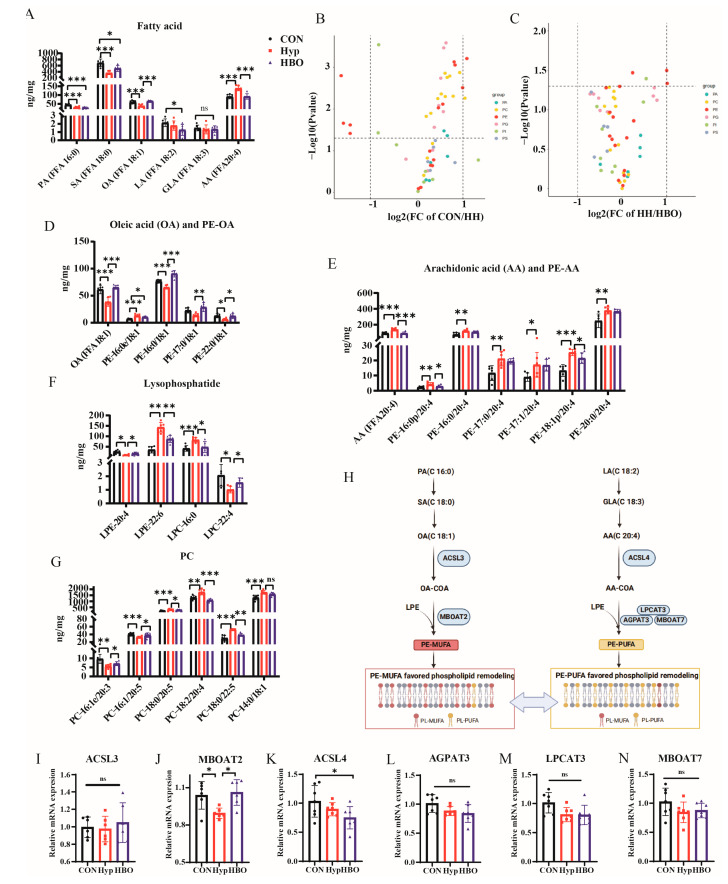
The histograms showed the absolute levels of lipids in the three groups by semi-target and target MS assay, including (**A**) Fatty acid. Volcano plots showing changes in PLs-OA in prefrontal cortex between (**B**) CON and Hypoxia group, (**C**) Hypoxia vs. HBO group. The vertical dotted line represented the |log2FC|≥ 1 threshold, while the horizontal dotted line represented the *p* value < 0.05 threshold. The significance of −log10 (*p* value) by *t*-test. Semi-target and target MS assay of (**D**) Oleic acid (OA) and PE-OA (**E**) Arachidonie acid (AA) and PE-AA (**F**) Lysophosphatide (**G**) PC (n = 6). (**H**) Schematic showing the synthesis and conversion into PLs of free fatty acids. (**I**,**J**) The mRNA levels of ACSL3, MBOAT2 in the prefrontal cortex (n = 6). (**K**–**N**) The mRNA levels of ACSL4, AGPAT3, LPCAT3, MBOA7 in the prefrontal cortex (n = 7). Significant differences were tested by one-way ANOVA. Significant level: ns: not statistically significant, * *p* < 0.05; ** *p* < 0.01; *** *p* < 0.001.

Given the significant change in FAs pathways observed in the KEGG analysis with OA showing the most pronounced differences between groups in the volcano plot, the correlative content in prefrontal cortex samples was accurately determined using UHPLC-MS/MS lipidomic analysis. The contents of OA and related raw materials palmitic acid (PA) and stearic acid (SA) were quantitatively measured. Only OA, a monounsaturated fatty acid (MUFA), decreased in the hypoxia group and increased post-HBO exposure (Figure 3A). Additionally, based on the results of untargeted UHPLC-MS analysis, PLs, triacylglycerol (TG), and diacylglycerol (DG) containing arachidonic acid (AA) changed significantly among the groups, so the contents of AA and related raw materials linoleic acid (LA) and gamma-linolenic acid (GLA) were quantitatively measured. Only AA, a polyunsaturated fatty acid (PUFA), a component that eventually incorporate into cell membranes showed significant increased in hypoxia group and decreased after exposed to HBO (Figure 3A). The balance between pro-ferroptotic PLs containing PUFA, which are highly susceptible to peroxidation, and anti-ferroptotic PLs containing MUFA, which exhibit antioxidant properties, plays a crucial role in determining FPT sensitivity [53,54]. Therefore, the content of related PLs was analyzed specifically. Analysis revealed that phosphatidylethanolamines (PEs) were the most predominant class (Figure 3B,C), with PE-OA decreasing under hypoxia and increasing post-HBO (Figure 3D), while PE-AA showed the opposite trend (Figure 3E). In agreement with this competition model, the decrease of PE-OAs was very similar to the increase of PE-AAs that share the same backbone of lyso-PE (e.g., PE-16:0/18:1 vs. PE-16:0/20:4, PE-17:0/18:1 vs. PE-17:0/20:4). FA is incorporated into membrane via lysophosphatidylcholine (LPC) and lysophosphatidylethanolamines (LPE) to produce membrane PLs. The content of LPE and LPC in the three groups is shown in the Figure 3F. Additionally, previous studies have shown that in addition to PE, phosphatidylcholine (PC) also played an important role in membrane formation and lipid peroxidation [54]. PC containing AA and OA was analyzed, with PC-AA being more responsive to oxygen changes than PC-OA (Figure 3G). These findings indicated that oxygen levels significantly influenced the PLs composition in the prefrontal cortex. Hypoxia was found to enrich the membrane with polyunsaturated PLs, while HBO exposure resulted in an enrichment of monounsaturated PLs.

### 3.4. HBO Reversed the Descent of MBOAT2 Caused by Hypoxia in Mice

The activity of enzymes responsible for the incorporation of MUFA and PUFA into PLs plays a crucial role in membrane PLs remodeling [53]. Therefore, the influence of oxygen content on the activity of the related enzymes was explored. These enzymes, including acyl-CoA synthetase long chain (ACSL) and lysophospholipid acyltransferase (LPLAT) as showed in Figure 3H. OA activation is mediated by ACSL3, followed by its incorporation into PLs catalyzed by MBOAT2. AA is preferentially activated by ACSL4 and then incorporated into PLs through LPCAT3, MBOAT7, or AGPAT3 [55]. The mRNA expression levels of MBOAT2 in hypoxia mice was decreased than CON mice, but this reduction can be reversed by HBO (Figure 3J). This may explain the alteration in PE-OA content observed under hypoxia and HBO conditions. There was no change in mRNA levels of others (Figure 3I,K–N).

### 3.5. HBO Relieved FPT of Central Nerve Cell Caused by Hypoxia

**Figure 4:**
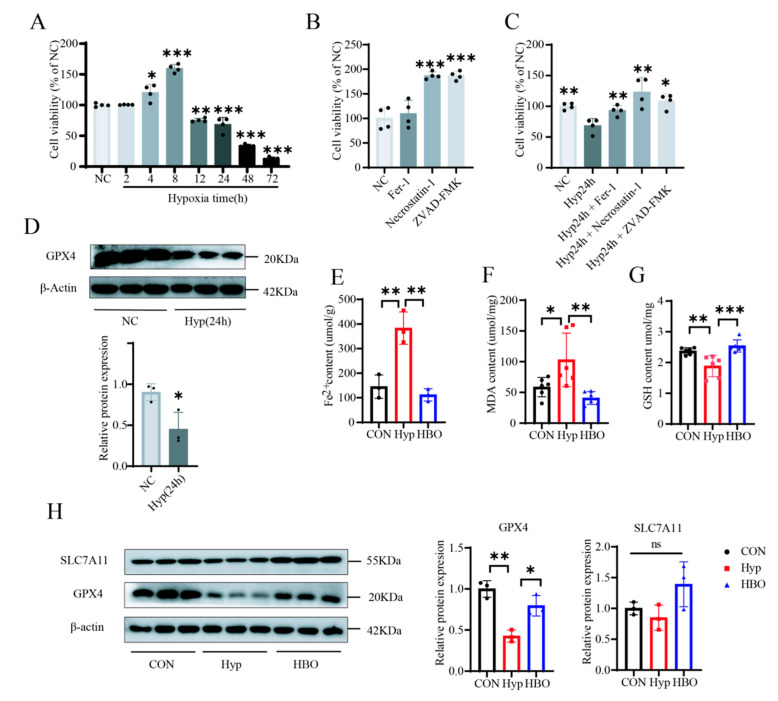
Oxygen content affected FPT in vivo and vitro. (**A**) Survival rate of SY5Y cells under different hypoxia time. (**B**) Cell survival under normoxia. (**C**) Cell survival under hypoxia (n = 4). (**D**) Western blots of GPX4 protein and corresponding quantitative results in SY5Y(n = 3). The level of (**E**) Fe^2+^ (**F**) MDA (**G**) GSH in the prefrontal cortex (n = 3–6). (**H**) Western blots of GPX4, SLC7A11 protein and corresponding quantitative results in the prefrontal cortex (n = 3). Significant differences were tested by one-way ANOVA Significant level: ns: not statistically significant, * *p* < 0.05; ** *p* < 0.01; *** *p* < 0.001.

In view of the above results, oxygen content caused membrane PLs remodeling. Hypoxia tended to enrich the membrane PLs in PUFA, while hyperoxia tended to enrich them in MUFA. And it is reported that PUFAs-containing membrane PLs are sensitive to lipid peroxidation and repeatedly associated with FPT. MUFA-containing PLs exhibited antioxidant properties and against FPT [56]. So whether changes in oxygen content affect FPT as predicted above was explored. In experiments conducted on SH-SY5Y cells, it was observed that cell survival initially increased when exposed to hypoxia for a duration of 2–8 h, which could be an indication of stress, then decreased from 12 h with the extent of survival decreasing in relation to the duration of hypoxia (Figure 4A). Hypoxia for 24 h was deemed sufficient to induce injury. In normoxia conditions, the survival rates of SH-SY5Y cells were increased by Necrostatin-1 (a necroptosis inhibitor) and ZVADFMK (an apoptotic inhibitor), but no significant improvement was observed when treated with Fer-1 (FPT inhibitor) (Figure 4B). However, under hypoxia conditions, Fer-1 led to an improvement in survival rates. WB results also showed that hypoxia decreased GPX4 expression in SY5Y cells (Figure 4D). This indicated that hypoxia would promote the occurrence of FPT in SY5Y cells.

To verify the above points to the sensitive spearhead of hypoxia-FPT, indicators of FPT in mouse cortical samples were examined including Fe^2+^ which contribute to excessive ROS and oxidative damage and biochemical markers MDA (indicating lipid peroxidation) and GSH (antioxidant defense). The results showed that FPT processed under hypoxia was blocked down by HBO, with increased GSH and reduced Fe^2+^ level, MDA (Figure 4E–G). The study also examined the role of SLC7A11, which mediates cystine uptake to enhance GSH synthesis and inhibit FPT [57]. Despite GSH changes, SLC7A11 levels remained consistent across all groups (Figure 4H), suggesting minimal impact of oxygen conditions on glutamic cystine transport. GPX4, an antioxidant defense enzyme that repairs lipids oxidative damage and inhibits FPT, was downregulated under hypoxia but restored under HBO (Figure 4H). These findings indicated that hypoxia triggered FPT in mice central nerve cells, which can be alleviated by HBO.

### 3.6. OA and MBOAT2 Induced PLs Remodeling in Neuronal Membrane Under Hypoxia in an Interdependent Manner, Alleviated the FPT

**Figure 5:**
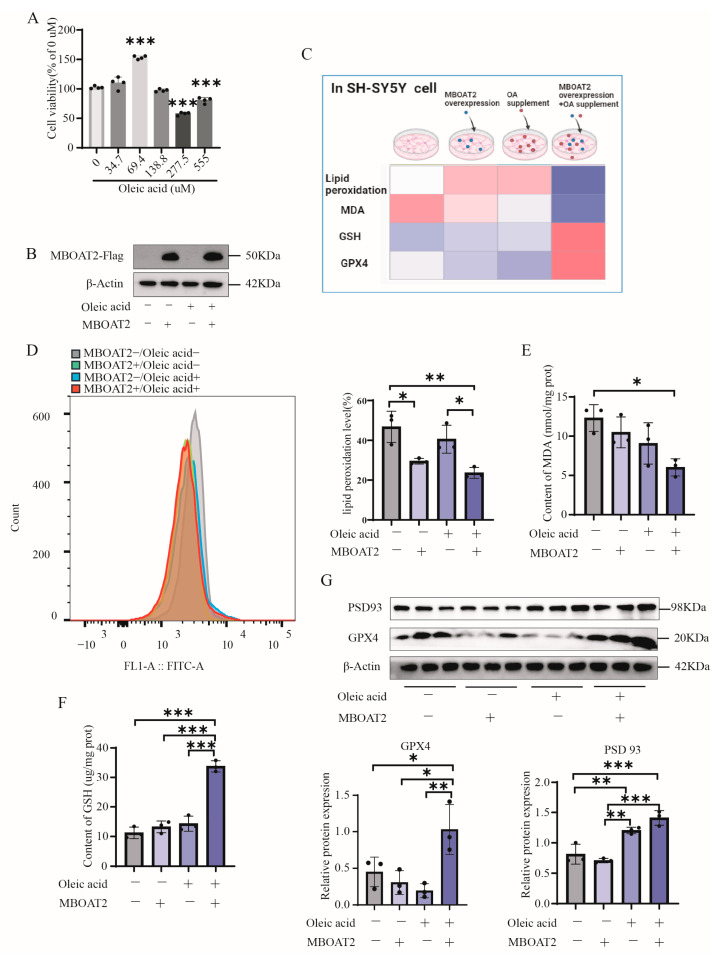
Effects of MBOAT2 and OA on FPT and cognitive markers. (**A**) Survival rate of SY5Y cells under different concentration of OA. (**B**) Effect of overexpression of MBOAT2 plasmid. (**C**) The heat map showed the expression of FPT indicators in each group. As it approached pink, the content was higher, while as it approached blue, the content was lower. Levels of (**D**) lipid peroxidation, (**E**) MDA, (**F**) GSH in SY5Y cell between different groups (n = 3). (**G**) Western blots of GPX4, PSD93 protein and corresponding quantitative results in cell (n = 3). Significant differences were tested by one-way ANOVA. Significant level: * *p* < 0.05; ** *p* < 0.01; *** *p* < 0.001.

The conclusion drawn from the study was that HBO can counteract the reduction of OA and MBOAT2 induced by hypoxia, consequently facilitating the remodeling of membrane phospholipids and mitigating ferroptosis of central nerve cells. The research focused on investigating the interrelationship between OA and MBOAT2. Initially, safe concentrations of OA were determined, with 70 μM was selected based on its protective effects on SY5Y cells in subsequent experiments (Figure 5A). Cells were categorized into four groups: hypoxia, MBOAT2 overexpression only, exogenous OA only, and simultaneous MBOAT2 overexpression with OA treatment. MBOAT2 expression levels varied across groups (Figure 5B). A heat map depicted FPT-related biomarker expression (Figure 5C). Notably, the group with simultaneous MBOAT2 overexpression and OA treatment exhibited reduced lipid peroxidation and MDA levels (Figure 5D,E), alongside increased GSH and GPX4 levels (Figure 5F,G), indicating resistance to FPT. Furthermore, there was a notable increase in PSD93 levels, indicating potential involvement of MBOAT2 and OA in synaptic function and memory processes (Figure 5G).

**Figure 6:**
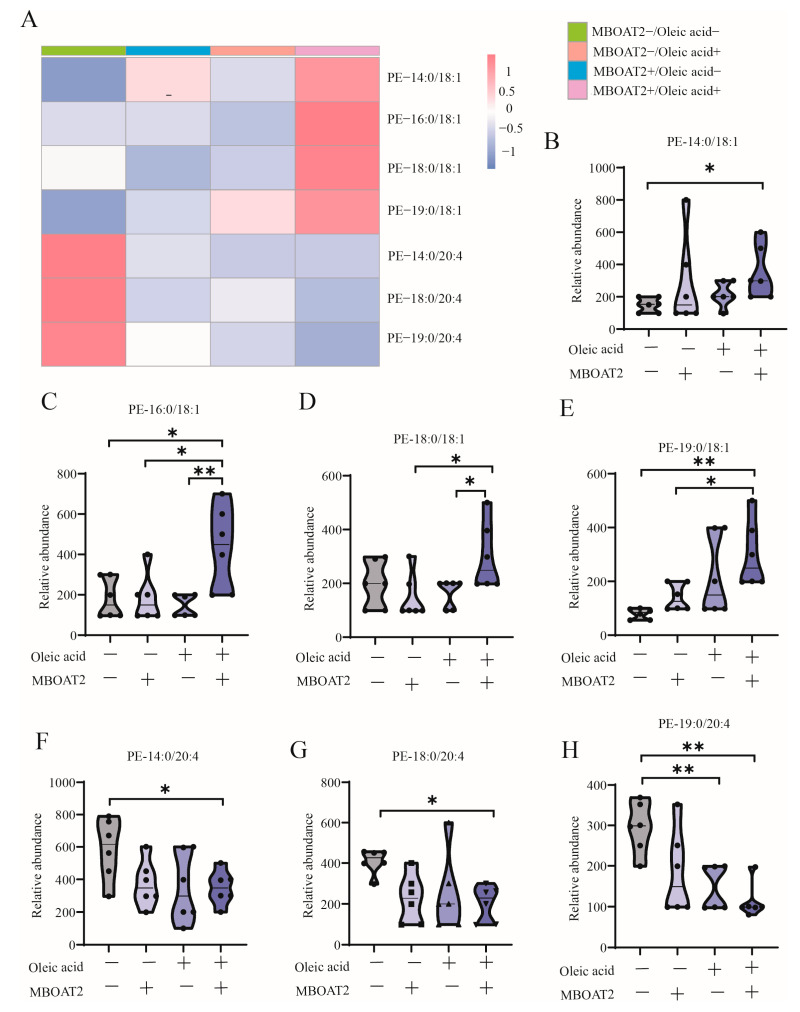
(**A**) The heat map showed relative levels of differential PLs, with red indicating higher relative levels and blue indicating lower relative levels. The histogram showed the absolute levels of PLs in the four groups by target MS assay, including (**B**) PE-14:0/18:1, (**C**) PE-16:0/18:1, (**D**) PE-18:0/18:1, (**E**) PE-19:0/18:1, (**F**) PE-14:0/20:4, (**G**) PE-18:0/20:4, (**H**) PE-19:0/20 (n = 6). Significant differences were tested by one-way ANOVA. Significant level: * *p* < 0.05; ** *p* < 0.01.

Given MBOAT2 catalyzed OA incorporation into PLs, the links between membrane PLs remodeling and FPT sensitivity [56], we hypothesize that MBOAT2 and OA competitively increase PL-MUFA levels, ultimately leading to a FPT-resistant cell state under hypoxia. Since MBOAT2 and OA inhibit FPT in a mutually dependent manner, the study sought to determine whether their interaction through PLs remodeling synergistically. To test this possibility, lipidomic analysis was performed in SY5Y cells (Figure 6A). The group that underwent simultaneous MBOAT2 overexpression and OA treatment demonstrated a noticeable increase in PE-OAs (Figure 6B–E), while other PE-AAs (Figure 6F–H) showed a significant decrease. These alterations in the composition of membrane phospholipids suggested that their contribution to FPT resistance under hypoxia.

## 4. Discussion

As the organ mostly dependent on oxygen, the brain consumed 20% of human resting O_2_ [58], physiological stressors related to hypoxia were prone to brain hypoxia and damage resulting in CI, making CI an urgent global health issue. HBO was known to alleviate brain hypoxia by increasing the partial pressure of oxygen in the blood and tissues. In this study, metabolomic and lipidomic analyses, and molecular biology, were used to study the effect and mechanism of hypoxia and HBO on cognitive function in mice. The results suggested that HBO alleviated CI and pathological injury of hypoxia mice by increasing the expression of OA and MBOAT2, thereby affecting the composition of membrane PLs and reducing hypoxia-induced central nerve cell FPT. In vitro cell experiments have confirmed that the two factors led to the remodeling of membrane PLs in an interdependent manner and affected the resistance of cells to FPT under hypoxia. Our limitation is that we have not further validated the role of oleic acid and MBOAT2 in CI of hypoxic mice.

The use of HBO in patient therapy dates back to the 17th century, and it remains the most effective method for supplying oxygen to the cells. During treatment, the environment in the HBO chamber causes the partial pressure of blood oxygen to rise and the solubility of blood oxygen in the plasma to increase significantly [59]. It had been proven beneficial in treating chronic wounds, osteomyelitis, bacterial infections, CO poisoning, and burns. Early studies from 1976 also explored its potential in CNS diseases [60], showing it can enhance nerve cell metabolism, lower intracranial pressure, and improve cognitive function and quality of life [61]. However, long-term use can lead to complications like middle ear injury, lung pressure damage, and hyperoxic myopia due to oxygen toxicity, limiting its widespread application [51,62]. Further research into its mechanisms underlying the observed effects aims to optimize its benefits and minimize risks.

Exogenous metabolites such as lipids, including OA, are potent modulators of cell function and fate [63]. OA, a MUFA commonly found in dietary oils, plays a crucial role as a major component of PLs in the brain’s cell membranes [64]. Its level and oxidation status are crucial for maintaining normal cell membrane integrity. Although the underlying mechanisms between OA and cognitive function have not been well elucidated, it was believed that OA was related to the onset of neurodegeneration disease [65], higher OA content was associated with lower risk of AD, MCI, and MCI-to-AD progression [66]. OA administration, converted in the gut to oleoylethanolamide and signaled to the brain via the autonomic nervous system, enhanced memory and counteracts cognitive decline by regulating neural stem and progenitor cell self-renewal and proliferation [67]. Therefore, it is highly likely that OA may be a beneficial nutrient that is closely related to neurological diseases. Human studies supported these benefits [68], and our research confirmed a consistent positive correlation between OA levels and cognitive function under varying oxygen conditions, highlighting OA’s potential as a beneficial nutrient for neurological health.

At present, studies on the inhibition of FPT by OA mainly focus on cancer cells [69,70,71], cultured mammalian cell lines [72,73], caenorhabditis elegans, and hepatocyte lipid peroxidation in mice [74], with the mechanism of OA reported to change the cellular lipid composition, characterized by decreased levels of polyunsaturated fatty acyl PLs and ether-linked PLs [72,73], though the molecular mechanisms remain unclear. OA does not consistently play a mitigating role in all pathological conditions associated with ferroptosis. It can exacerbate FPT in cardiac cells under a high-fat diet via STAT3/NCOA4-mediated ferritinophagy [65] and accelerate FA oxidation in skeletal muscle cells [75]. Therefore, it is necessary to explore the effect of OA on neuron FPT under oxygen content fluctuation.

OA’s activation by ACSL3 and its esterification with LPLs by MBOAT2 to form membrane PLs are key processes. The result showed oxygen content had a greater impact on MBOAT2 than ACSL3. Oxygen content fluctuations affected OA’s integration into the membrane, influencing FPT sensitivity, with membrane PLs playing a more critical role than free FAs [76]. The previous study reported that even with ACSL3 knockout, OA can exert protective effects at high concentrations, as other ACSL enzymes can activate MUFAs [73]. In hypoxic conditions, our findings revealed that there were no alterations in FPT-associated markers in cells lacking OA accompanied by MBOAT2 overexpression or cells with elevated levels of OA without MBOAT2 overexpression. However, the combination of OA supplementation and MBOAT2 overexpression effectively counteracted FPT, aligning with previous findings that MBOAT2, as a FPT inhibitor, requires either endogenous or exogenous MUFA [76]. Further test revealed that MBOAT2 and OA suppressed FPT by modulating phospholipid metabolism, particularly affecting PE. Accumulation of oxidized PLs, especially oxidized PE is known to be critical death signals and one of the key hallmark of FPT [53]. The lipidomics results showed that MBOAT2 catalyzed MUFA binding to PE, reducing PE-PUFA content, leading to an anti-FPT state.

## 5. Conclusions

The research investigated the potential protective effect of HBO therapy on CI induced by hypoxia. The findings indicated that HBO treatment mitigated CI and the associated pathological damage in hypoxic mice. HBO treatment upregulated the expression of both OA and MBOAT2, which synergistically led to membrane PLs remodeling, thereby reducing FPT and alleviating CI resulting from hypoxia. These results highlighted the interdependent role of OA and MBOAT2 in HBO’s effectiveness against hypoxia-induced CI, offering a new perspective for treating CI with HBO.

## Data Availability

The original contributions presented in the study are included in the article/Supplementary Material; further inquiries can be directed to the corresponding author.

## Supplementary Materials

The following supporting information can be downloaded at: https://www.mdpi.com/article/10.3390/antiox13111320/s1, Figure S1: Evaluation of metabolomics LC-MS analysis; Table S1: Primers used for qRT-PCR; Table S2: Differential metabolites in metabolomics. Table S3: Differential metabolites in lipidomics.
