# Supplementary material for: Hyperbaric Oxygen Improves Cognitive Impairment Induced by Hypoxia via Upregulating the Expression of Oleic Acid and MBOAT2 of Mice
Source: Antioxidants (Basel). 2024 Oct 29;13(11):1320. doi: 10.3390/antiox13111320 (PMC11591255; doi:10.3390/antiox13111320)
Supplement: Supplementary file 1 [file antioxidants-13-01320-s001.zip › antioxidants-3255819-supplementary.pdf]

Supplementary material

Hyperbaric oxygen improves cognitive impairment induced by hypoxia via upregulating the expression of oleic acid and MBOAT2 of mice

Zhen Li [1,2,†], Jun Fu [1,3,†], Kaiyuan Jiang [1], Jie Gao [1], Yuejun Guo [1], Chen Li [1], Liangcai Zhao [1], Jutaek Nam [2,*], Hongchang Gao [1,3,*]

[1] School of Pharmaceutical Sciences, Oujiang Laboratory (Zhejiang Lab for Regenerative Medicine, Vision and Brain Health); Institute of Metabonomics & Medical NMR, Wenzhou Medical University, Wenzhou 325035, China

[2] College of Pharmacy, Chonnam National University, Gwangju 61186, Korea

[3] Innocation Academy of Testing Technology, Research and experiment center， Wenzhou Medical University

***Corresponding authors at:** College of Pharmacy, Chonnam National University, Gwangju 61186, Korea.

Email: namj@jnu.ac.kr (Jutaek Nam)

School of Pharmaceutical Sciences; Oujiang Laboratory; Institute of Metabonomics & Medical NMR, Wenzhou Medical University, Wenzhou 325035, China.

Email: gaohc27@wmu.edu.cn (Hongchang Gao)

[†] These authors have contributed equally to this work.

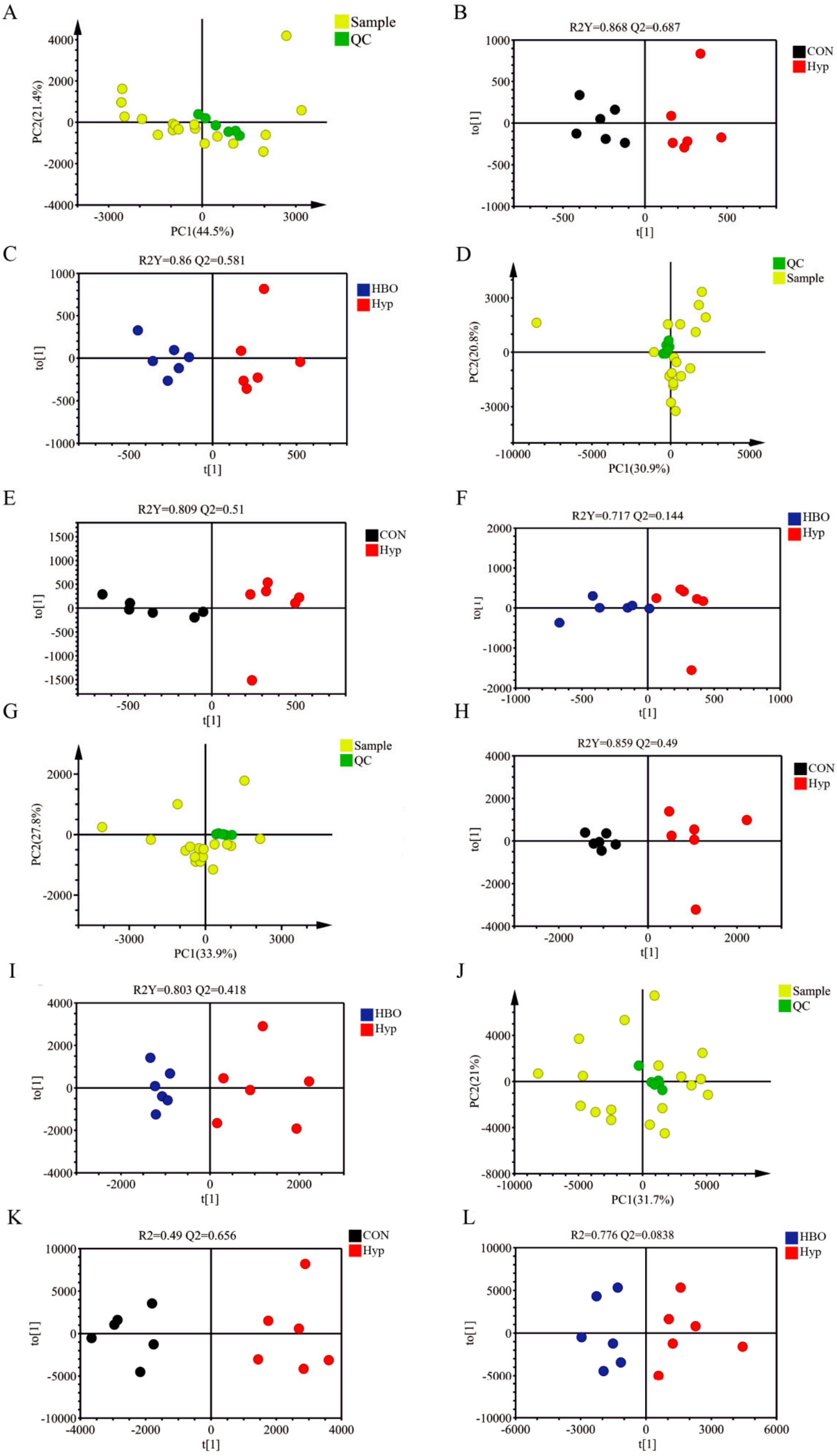

A
Sample
QC
PC2(21.4%)
PC1(44.5%)
B
R2Y=0.868 Q2=0.687
CON
Hyp
to[1]
t[1]
C
R2Y=0.86 Q2=0.581
HBO
Hyp
D
QC
Sample
PC2(20.8%)
PC1(30.9%)
E
R2Y=0.809 Q2=0.51
CON
Hyp
F
R2Y=0.717 Q2=0.144
HBO
Hyp
G
Sample
QC
PC2(27.8%)
PC1(33.9%)
H
R2Y=0.859 Q2=0.49
CON
Hyp
I
R2Y=0.803 Q2=0.418
HBO
Hyp
J
Sample
QC
PC2(21%)
PC1(31.7%)
K
R2=0.49 Q2=0.656
CON
Hyp
L
R2=0.776 Q2=0.0838
HBO
Hyp

Figure S1. Evaluation of metabolomics LC-MS analysis (A) The QC samples (green square marker) were evaluated in PCA scores plots (B-C) OPLS-DA under Amide negative mode. (D-F) Amide positive mode (n = 6). Evaluation of lipidomics LC-MS analytical under (G-I) C18 negative mode. (J-L) C18 positive mode (n = 6).

Table S1 Primers used for qRT-PCR.

gene accession number	Primer	Sense/antisense	Sequence
NM_001321265.2	MBOAT2	FORWARD	AGTTGTTCCGTTTGTGCTCCTCTC
		REVERSE	GCAGCAACAGCAACACCAAGATAC
NM_001354158.2	ACSL3	FORWARD	CGAATCGGCTACTCTTCACCACAG
		REVERSE	AGTGTTGGCTTCAGCACGGATG
NM_001404870.1	AGPAT3	FORWARD	ATTGGCTTCGTCTTCGTGGTGAG
		REVERSE	GCGGTATAGGTGCTTGCTGATGG
NM_145130.3	LPCAT3	FORWARD	CTACCCGTTGGCTCTGTTTTACCG
		REVERSE	ACTGAAGCACGACACATAGCAAGG
NM_001146056.3	MBOAT7	FORWARD	GCTGCTGAGGGCTATTTGGAGTC
		REVERSE	GTAGGCACGCATCTTCAGGAACC
NM_001318509.2	ACSL4	FORWARD	CCGCTTGTGACTTTATATGCTACCC
		REVERSE	GCCGCCTTCAGTTTGCTTTCC

Table S2 Differential metabolites in metabolomics.

Meatabolites	Mass(m/z)	RT (min)	Formula	Adducts	HH vs CON			HBO vs HH		
					log2(FC)	P-Value	VIP	log2(FC)	P-Value	VIP
Hypoxanthine	135.0305	6.69	$C_5H_4N_4O$	[M-H]-	-0.68	0.0001	3.09	0.62	0.0198	2.81
Xanthine	151.0245	6.97	$C_5H_4N_4O_2$	[M-H]-	-1.06	0.0150	2.90	0.76	0.0029	2.25
Ribose 5-phosphate	229.0085	10.30	$C_5H_{11}O_8P$	[M-H]-	-0.45	0.0468	3.11	0.37	0.0454	2.72
Guanosine	282.0824	8.02	$C_{10}H_{13}N_5O_5$	[M-H]-	-1.27	0.0475	5.11	0.72	0.0420	2.91
Uridine	243.0623	6.72	$C_9H_{12}N_2O_6$	[M-H]-	-0.53	0.0316	2.67	0.48	0.0121	2.57
Inosine	267.0730	7.34	$C_{10}H_{12}N_4O_5$	[M-H]-	-0.57	0.0037	15.97	0.52	0.0046	14.66
Creatine	132.0766	9.41	$C_4H_9N_3O_2$	[M+H]+	-0.36	0.0008	4.07	0.22	0.0415	3.85
Lactic acid	89.0241	5.46	$C_3H_6O_3$	[M-H]-	-0.58	0.0005	7.52	0.49	0.0070	6.63
l-phenylalanine	120.0801	7.59	$C_9H_{11}NO_2$	[M+NH4]+	-0.55	0.0021	3.03	0.33	0.0463	2.56
Sarcosine	88.0398	9.31	$C_3H_7NO_2$	[M-H]-	-0.27	0.0420	1.25	0.29	0.0188	1.33
S-Adenosylhomocysteine	383.1109	10.09	$C_{14}H_{20}N_6O_5S$	[M-H]-	-0.66	0.0366	1.11	0.57	0.0085	1.01
Pyroglutamic acid	128.0340	9.56	$C_5H_7NO_3$	[M-H2O-H]-	-0.73	0.0004	1.53	0.69	0.0364	1.45
N-Acetyl-L-glutamic acid	188.0551	6.16	$C_7H_{11}NO_5$	[M-H]-	-0.38	0.0030	1.20	0.40	0.0130	1.31
N-Acetyl-L-alanine	130.0510	6.45	$C_5H_9NO_3$	[M-H]-	-0.27	0.0466	2.07	0.29	0.0249	2.18
N-Acetyl-L-aspartic acid	174.0399	6.46	$C_6H_9NO_5$	[M-H]-	-0.32	0.0386	2.83	0.38	0.0094	3.13
Sinapoyl malate-4'-methyl ester	427.0777	6.72	$C_{16}H_{18}O_9$	[M+Na-2H]-	-0.52	0.0079	1.83	0.65	0.0176	2.16
Succinate	248.9709	9.59	$C_4H_6O_4$	[M-H2O-H]-	-0.65	0.0007	1.11	0.69	0.0022	1.13
Glycocholic acid	464.3121	5.71	$C_{26}H_{43}NO_6$	[M-H]-	-0.64	0.0009	1.27	0.84	0.0000	1.51
Palmitic acid	255.2316	3.22	$C_{16}H_{32}O_2$	[M-H]-	-0.62	0.0004	1.15	0.60	0.0054	1.12
Oleic acid	281.2469	3.20	$C_{18}H_{34}O_2$	[M-H]-	-1.24	0.0001	4.27	0.79	0.0056	3.10
Eicosenoic acid	309.2790	3.15	$C_{20}H_{38}O_2$	[M-H]-	-1.90	0.0009	1.59	1.28	0.0001	1.16

Docosahexaenoic acid	327.2342	3.21	$C_{22}H_{32}O_2$	[M-H]-	-1.33	0.0018	11.76	1.02	0.0137	9.76
Docosatetraenoic acid	331.2639	3.17	$C_{22}H_{36}O_2$	[M+Na-2H]-	-1.39	0.0010	3.21	1.07	0.0087	2.61
FA 30:6	219.1745	1.28	$C_{30}H_{48}O_2$	[M-2H]2-	-0.40	0.0000	1.93	0.19	0.0073	1.13
PG(36:4)	769.4976	5.46	$C_{42}H_{75}O_{10}P$	[M-H]-	-0.60	0.0005	2.14	0.86	0.0019	2.61
PG(38:5)	795.5172	5.45	$C_{44}H_{77}O_{10}P$	[M-H]-	-0.69	0.0005	1.78	0.78	0.0015	1.81
PG(40:8)	817.5055	5.42	$C_{46}H_{75}O_{10}P$	[M-H]-	-0.42	0.0035	1.35	0.48	0.0134	1.48
PG(44:12)	865.5057	5.41	$C_{50}H_{75}O_{10}P$	[M-H]-	-0.37	0.0054	4.90	0.38	0.0076	5.14
PS(40:7)	832.5123	5.61	$C_{46}H_{76}NO_{10}P$	[M-H]-	-0.69	0.0007	1.78	0.75	0.0033	1.88
PS O-42:5	896.6010	5.66	$C_{48}H_{86}NO_9P$	[M+Formate]-	-0.26	0.0150	2.24	0.30	0.0178	2.25
PS(42:10)	854.4997	5.59	$C_{48}H_{74}NO_{10}P$	[M-H]-	-0.53	0.0027	1.08	0.52	0.0212	1.20
PS 44:10	882.5286	5.58	$C_{50}H_{78}NO_{10}P$	[M-H]-	-0.32	0.0130	1.90	0.22	0.0372	1.54
PS(44:12)	878.5002	5.58	$C_{50}H_{74}NO_{10}P$	[M-H]-	-0.55	0.0018	2.29	0.54	0.0099	2.43
PI(36:4)	857.5197	5.66	$C_{45}H_{79}O_{13}P$	[M-H]-	-0.54	0.0055	1.84	0.67	0.0032	2.20
PI(38:5)	883.5353	5.50	$C_{47}H_{81}O_{13}P/C_{52}H_{72}O_8$	[M-H]-/[M+OAc]-	-0.68	0.0011	2.94	0.40	0.0298	1.48
PI(38:6)	881.5185	5.41	$C_{47}H_{79}O_{13}P$	[M-H]-	-0.63	0.0007	1.21	0.70	0.0007	1.38
PC(17:0/12-hete)	870.5835	5.51	$C_{45}H_{82}NO_9P$	[M+CH3COO]-	-0.91	0.0011	2.54	0.84	0.0038	2.31
PC 38:4;O/PS O-40:4	870.5835	5.51	$C_{46}H_{84}NO_9P$	[M+Formate]-	-0.60	0.0003	2.28	0.40	0.0030	1.59
PA O-30:2/CerPE 30:2;O2	199.8040	7.90	$C_{33}H_{63}O_7P/C_{32}H_{63}N_2O_6P$	[M-3H]3-	-0.17	0.0107	1.09	0.23	0.0040	1.13
SHexCer 43:6;O2	894.5813	5.50	$C_{49}H_{85}NO_{11}S$	[M-H]-	-0.64	0.0002	1.94	0.41	0.0047	1.21
LysoPE(0:0/16:0)	436.2800	5.76	$C_{21}H_{44}NO_6P$	[M-H]-	-0.68	0.0032	1.61	0.77	0.0004	1.65
LPE(18:1)	480.3082	5.81	$C_{23}H_{46}NO_7P$	[M+H]+	-0.30	0.0028	2.14	0.31	0.0289	2.57
LysoPE(0:0/20:1)	506.3221	5.75	$C_{25}H_{50}NO_7P$	[M-H]-	-0.34	0.0298	1.26	0.62	0.0017	1.91
LysoPE(0:0/20:0)	508.3317	5.73	$C_{25}H_{52}NO_7P$	[M-H]-	-0.45	0.0159	1.11	0.64	0.0003	1.35
LysoPE(0:0/22:6)	524.2979	5.75	$C_{27}H_{44}NO_7P$	[M-H]-	-0.42	0.0043	1.10	0.59	0.0019	1.34
LysoPC(16:0/0:0)	540.3298	5.78	$C_{24}H_{50}NO_7P$	[M+Na-2H]-	-0.39	0.0285	5.65	0.57	0.0081	7.51

LPC(18:1)	566.3440	5.75	$C_{26}H_{52}NO_7P$	[M+HCOO]-	-0.39	0.0061	3.22	0.68	0.0040	4.69

Table S3 Differential metabolites in lipidomics

Meatabolites	Mass(m/z)	RT (min)	Formula	Adducts	HH vs CON			HBO vs HH		
					log2(FC)	P-Value	VIP	log2(FC)	P-Value	VIP
FA 18:4;O	145.0970	11.69	$C_7H_{14}O_3$/$C_{18}H_{28}O_3$	[M-H]-	0.43	0.0167	1.29	-0.52	0.0083	1.11
FA 14:8;O2	303.0839	8.40	$C_{14}H_{12}O_4$	[M+OAc]-	0.57	0.0438	3.95	-0.64	0.0105	3.22
FOH 16:10;O4	285.0709	8.75	$C_{16}H_{14}O_5$	[M-H]-	0.55	0.0320	2.28	-0.47	0.0313	1.44
PC(18:0/22:4)	838.6324	9.43	$C_{48}H_{88}NO_8P$	[M+H]+	0.34	0.0001	1.76	-0.14	0.0479	1.42
PC(18:3/22:6)	828.5461	7.79	$C_{48}H_{78}NO_8P$	[M+H]+	0.38	0.0003	1.33	-0.16	0.0323	1.01
PC(20:3/20:4)	832.5789	7.89	$C_{48}H_{82}NO_8P$	[M+H]+	0.39	0.0072	1.86	-0.26	0.0347	1.51
PC(20:4/20:4)	830.5640	8.08	$C_{48}H_{80}NO_8P$	[M+H]+	0.42	0.0002	1.09	-0.21	0.0118	1.04
PC(32:1)	754.5301	7.76	$C_{40}H_{78}NO_8P$	[M+Na]+	0.50	0.0015	2.36	-0.20	0.0474	1.50
PC O-46:7|PC O-18:3_28:4	902.7287	12.25	$C_{54}H_{96}NO_7P$	[M+H]+	0.61	0.0002	2.01	-0.22	0.0485	1.42
PE(P-18:0/20:4)	752.5723	7.69	$C_{43}H_{78}NO_7P$	[M+H]+	0.41	0.0006	1.16	-0.23	0.0103	1.18
SL 16:3;O/24:6	668.4250	3.09	$C_{40}H_{63}NO_5S$/$C_{36}H_{64}NO_8P$	[M-H]-	2.40	0.0377	1.07	-2.15	0.0441	1.10
SL 13:1;O/30:5;O	751.5683	7.69	$C_{43}H_{75}NO_6S$	[M+NH4]+	0.44	0.0009	1.75	-0.25	0.0232	1.70
Sphingomyelin	829.7215	12.48	$C_{48}H_{97}N_2O_6P$	[M+H]+	0.47	0.0074	1.28	-0.31	0.0405	1.35
LPI 22:4/PG 25:4;O2	323.1530	4.06	$C_{31}H_{53}O_{12}P$	[M-2H]2-	1.39	0.0141	1.01	-1.20	0.0200	1.00
AHexCer (O-18:3)12:1;O2/12:0;O	818.6153	11.27	$C_{48}H_{85}NO_{10}$	[M+H-H2O]+	0.35	0.0001	3.51	-0.21	0.0043	2.78
DG(16:0/20:4)	634.5381	9.30	$C_{39}H_{68}O_5$	[M+NH4]+	0.37	0.0073	1.25	-0.46	0.0017	1.61
DG(36:1)	645.5438	9.83	$C_{39}H_{74}O_5$	[M+Na]+	0.44	0.0114	3.15	-0.60	0.0010	4.34
DG(18:1/20:4)	660.5559	9.43	$C_{41}H_{70}O_5$	[M+NH4]+	0.42	0.0176	1.15	-0.58	0.0043	1.72
DG(18:0/20:4)	662.5717	9.85	$C_{41}H_{72}O_5$	[M+NH4]+	0.36	0.0383	6.25	-0.65	0.0018	10.08
DG(38:4)	667.5262	9.85	$C_{41}H_{72}O_5$	[M+Na]+	0.72	0.0063	2.99	-0.75	0.0047	4.18
DG(18:0/22:6)	686.5709	9.74	$C_{43}H_{72}O_5$	[M+NH4]+	0.58	0.0126	1.62	-0.41	0.0461	1.16

DG(40:4)	695.5541	10.19	$C_{43}H_{76}O_5$	[M+Na]+	0.45	0.0036	1.25	-0.28	0.0054	1.15
TG(50:9)	839.6328	9.43	$C_{53}H_{84}O_6$	[M+Na]+	0.35	0.0000	1.34	-0.15	0.0109	1.06
TG(16:0/16:0/18:1)	855.7370	12.54	$C_{53}H_{100}O_6$	[M+Na]+	0.59	0.0001	1.93	-0.28	0.0020	1.59
TG(16:0/18:0/18:1)	883.7742	12.77	$C_{55}H_{104}O_6$	[M+Na]+	0.55	0.0001	1.69	-0.18	0.0266	1.25
TG(16:0/16:0/22:6)	901.7236	12.27	$C_{57}H_{98}O_6$	[M+Na]+	0.59	0.0003	2.54	-0.22	0.0457	1.86
TG(16:0/18:1/20:4)	903.7360	12.37	$C_{57}H_{100}O_6$	[M+Na]+	0.63	0.0000	1.57	-0.24	0.0027	1.09
TG(16:0/18:0/20:4)	905.7568	12.59	$C_{57}H_{102}O_6$	[M+Na]+	0.55	0.0011	1.41	-0.24	0.0212	1.24
TG(16:0/18:0/22:6)	929.7536	12.52	$C_{59}H_{102}O_6$	[M+Na]+	0.52	0.0000	2.20	-0.17	0.0455	1.35
TG(18:0/18:1/20:4)	931.7657	12.61	$C_{59}H_{104}O_6$	[M+Na]+	0.55	0.0000	1.90	-0.14	0.0432	1.15
TG(16:0/20:4/22:6)	949.7282	12.09	$C_{61}H_{98}O_6$	[M+Na]+	0.63	0.0000	1.60	-0.17	0.0479	1.08
TG(18:0/20:4/22:6)	977.7506	12.37	$C_{63}H_{102}O_6$	[M+Na]+	0.65	0.0000	1.58	-0.19	0.0081	1.12
Anserine/ST 19:2;O3;GlcA	239.1130	11.65	$C_{10}H_{16}N_4O_3$/$C_{25}H_{36}O_9$	[M-H]-	0.46	0.0248	2.63	-0.61	0.0096	2.71
Adenosine 5'-monophosphate	346.0520	14.00	$C_{10}H_{14}N_5O_7P$	[M-H]-	0.44	0.0176	2.15	-0.41	0.0224	1.82
Glutarylcarnitine	276.1433	8.01	$C_{12}H_{21}NO_6$	[M+H]+	1.71	0.0092	2.59	-0.91	0.0389	2.36
